# Geographic trends of tobacco-related cancers in Cyprus

**DOI:** 10.1186/s12971-015-0048-5

**Published:** 2015-07-31

**Authors:** Paraskevi Farazi, Lina Lander, Pavlos Pavlou, Katherine Watkins, Lynne Le, Amr Soliman

**Affiliations:** Department of Life and Health Sciences, University of Nicosia, 46 Makedonitissas Ave, P.O. Box 24005, Nicosia, 1700 Cyprus; Mediterranean Center for Cancer Research, 46 Makedonitissas Ave, P.O. Box 24005, Nicosia, 1700 Cyprus; Department of Epidemiology, 984395 University of Nebraska Medical Center, Omaha, NE 68198-4395 USA; Cyprus Cancer Registry, Ministry of Health, Corner of Prodromou 1 and Chilonos 17, 1448 Nicosia, Cyprus

**Keywords:** Cyprus, Tobacco, Cancer, Geographic, Trend, Epidemiology

## Abstract

**Background:**

Causal relationships have been previously established between smoking and various cancers. In Cyprus, 39 % of men and 14 % of women reported daily smoking in 2008. The objective of this study was to compare the incidence of tobacco-related cancers to all other cancers by district and rural–urban classification to understand the impact of tobacco in Cyprus.

**Methods:**

Data on lung, urinary bladder, oral, pharyngeal, head/neck, and laryngeal cancers were obtained from the Cyprus Cancer Registry (1998–2008). There were 3,635 patients with tobacco-related cancers and 18,780 with non-tobacco cancers. Univariate analysis comparing tobacco-related cancers and all other cancers were conducted with regards to age at diagnosis, age groups, sex, smoking status, disease stage, and rural/urban status, with a *p*-value of 0.05 considered significant. Smoking prevalence, lung cancer, and bladder cancer rates of Cyprus were also compared to a number of other European countries.

**Results:**

Patients with tobacco-related cancers were older than those with non-tobacco cancers (mean age 67.2 ± 12.4 vs. 62.4 ± 17.1, *p* < 0.0001). Among those with tobacco-related cancers, 80.1 % were male compared to 45.4 % males with other cancer types. The proportion of ever smokers was higher among males compared to females in urban and rural districts. Sub-districts 41 (Age Adjusted Rate (AAR) 41.9, 95 % CI: 35.7-48.1), 60 (AAR 40.3, 95 % CI: 35.2-45.3), and 50 (AAR 36.3, 95 % CI: 33.8-38.7) had the highest rates of tobacco-related cancers. The overall tobacco-related cancer rate was the highest among males in urban districts (AAR 60.8, 95 % CI: 58.2-63.5). Among tobacco-related cancers, lung cancer had the highest overall AAR (17.9 per 100,000) while head and neck cancer had the lowest overall AAR (5.3 per 100,000). Additionally, even though Cypriot males aged 65–69 years old exhibited higher smoking prevalence than other European countries, the overall lung and bladder cancer rates were lower in Cyprus.

**Conclusion:**

Despite the high proportion of smokers in Cyprus, cancer rates are low compared to other countries. Future in-depth measurements of relevant risk factors and smoking exposure can help understand this phenomenon and provide insights for cancer prevention.

## Introduction

Tobacco smoking represents the most preventable cause of morbidity and mortality worldwide [1, 2]. It directly causes approximately 5 million deaths globally every year, and indirectly, through the effects of second-hand smoke, an additional 600,000 deaths [3]. These trends are increasing and it is projected that tobacco will be responsible for the death of 8 million people annually by the year 2030 [1, 4, 5]. Furthermore, mortality attributed to tobacco has been increasing at a higher rate in developing countries, with higher rates among men compared to women [2].

Tobacco smoke contains approximately 4,000 potentially noxious chemicals, of which more than 69 have been classified as carcinogens [6, 7]. Smoking accounts for about 74 % of trachea, bronchus, and lung cancers, in addition to a number of other cancers [1, 4, 5, 7]. Associations with tobacco smoking have also been found with liver, ureter, nasal cavity and sinus cancers [8]. Tobacco smoking is also associated with an increased risk of other diseases including raised rates of ischemic heart disease and stroke, diseases of the respiratory system, and communicable diseases [1, 6, 9, 10].

Tobacco smoking prevalence is increasing in many parts of the world, including Cyprus, a small island in the Eastern Mediterranean. The morbidity and mortality attributable to tobacco can be estimated using several models [1, 2, 11–13]. According to the 2004 World Health Organization (WHO) estimates for adult Cypriots aged 30 years and over, nearly 10 % of annual deaths are attributable to tobacco [2]. Furthermore, one-fifth of malignant neoplasm deaths are due to tobacco consumption; this proportion increases to one-quarter when only considering those who are 60–69 years of age [2]. Tobacco is also responsible for nearly three-quarters of deaths from trachea, bronchus and lung cancers [2].

To date there has not been systematic data collection on the smoking prevalence in Cyprus [10, 14]. Surveys conducted between 1989 and 2008 found that among male respondents, smoking prevalence has been relatively stable (between 38-43 %) [14–19]. However, prevalence doubled among females, from 7 % in 1989 to 14 % in 2008 [14–19]. Adolescent smoking rates in Cyprus have also been investigated and are comparable to other European and eastern Mediterranean countries. In Cyprus middle schools (12–15 years old), 6-10 % of the students smoke regularly and 19-28 % have tried smoking [14, 19]. Among high school students, approximately one-quarter smoke regularly and approximately half have tried smoking [14, 19]. The numbers increase even further when examining only the 17–18 age groups [19]. Rates for other countries can range from 5 % in Greece (both boys and girls 11–15 year olds) to 8 % (boys) and 11 % (girls) in the United Kingdom of the same age [10, 14, 19].

Although cancer incidence in Cyprus may be lower than that of neighboring countries, it has slightly increased between 1998 and 2008 [20], which suggests that the high proportion of individuals who partake in cigarette or other forms of smoking can eventually lead to higher rates of cancer in the coming years due to the lag period between tobacco exposure and disease onset. The objective of this study was to compare the incidence of several tobacco-related cancers to the incidence of all other cancers by district and by rural–urban classification and to other European countries to better understand the impact of tobacco in Cyprus.

## Methods

### Ethical consent

The use of data in this study was approved by the Cyprus National Bioethics Committee, reference number ΕΕΒΚ ΕΠ 2012.01.26.

### Data sources

**Fig. 1:**
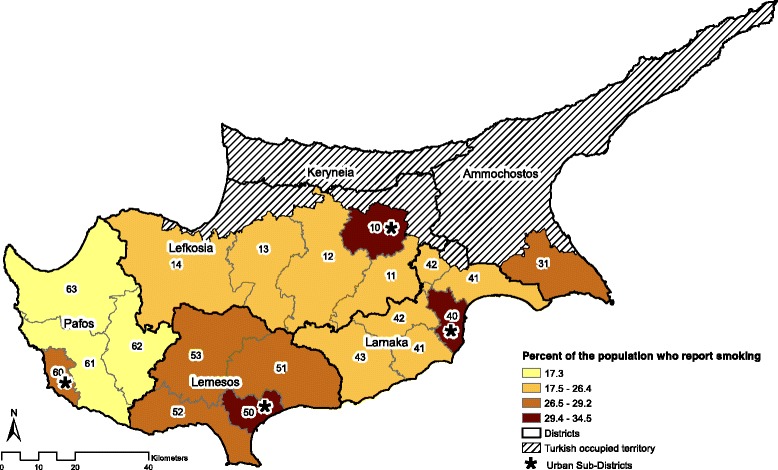
Proportion of smokers in the general population by urban and rural areas of sub-districts

The Cyprus Cancer Registry (CyCR) is a population-based cancer registry established in 1998 with the support from the Middle East Cancer Consortium (MECC) and the National Cancer Institute, USA. The CyCR includes the government-controlled southern part of the island of Cyprus and is administered by the Cyprus Ministry of Health (MOH). Patients who are permanent residents of the area not under the effective control of the government of the Republic of Cyprus (labelled as Turkish occupied territory in Fig. 1) were not included in the analyses. Using the classification system established by MECC, the CyCR uses ICD-O-3 to record information on 57 different cancer types, and the CANREG4 software, used for registration, derives the appropriate ICD-10 codes [21, 22]. MECC conducted an audit in 1999 and found that the CyCR accomplished 88 % case coverage. Since then, the registry has improved, and based on a 2008 case finding evaluation, the CyCR is estimated to accomplish 95 % coverage of all cancer cases in government-controlled Cyprus [22].

Data for this study were obtained from the CyCR and contained a unique identifier (that was created and kept by the Health Monitoring Unit (HMU)) and included the year of diagnosis, sex, and age at diagnosis. In addition, smoking data was obtained from the registry, albeit it was not available for all patients. The missing smoking information from the Cyprus registry included in this study is comparable to the rates reported from other cancer registries in the region [23]. Cancer data were available for years 1998 – 2008. Annual age and sex-specific population estimates by geographic region were derived through linear regression analyses of the population census data of 1992, 2001, and 2011 available from the Cyprus statistical service. Due to a large population increase between 2001 and 2011, growth estimates rates were calculated independently between 1992 to 2001 and 2001 to 2011. Data on smoking prevalence by urban/rural area of residence was obtained from the Cyprus statistical service who collected this information as part of their 2008 European Health Survey.

There are 5 districts in Cyprus: Ammochostos, Larnaka, Lefkosia, Lemesos, and Pafos. Kyreneia as shown in Fig. 1 belongs to the Turkish occupied territory and therefore is not considered a district of Cyprus. Each district is comprised of multiple municipalities, which are each assigned a 4 digit code by the Cyprus statistical service. We collapsed these municipalities according to the first 2 digits of the code into 18 sub-districts, which served as the geographic areas for the cancer incidence analyses. All districts contain urban and rural sub-districts, except Ammochostos, which is considered primarily rural (Fig. 1).

Data used for the comparison with other European countries was extracted from Cancer Incidence in Five Continents Plus (CI5 Plus) from the International Agency for Research on Cancer [24]. Lung and bladder cancer rates were from the years 1997–2007, which is similar to the time period used in this study (1998–2008). Further, the cancer rates for Cyprus used in the comparison with other countries were derived from the present study, not from CI5. Information regarding smoking prevalence for the years 1990 and 2000 was extracted from a published article by Ng et. al. [25]. These years were chosen due to the lag period between tobacco exposure and cancer onset. Although a large number of cancers are related to tobacco and smoking, our study is limited to three main tobacco-related cancers: head/neck and oral, lung, and urinary/bladder cancers.

### Statistical analysis

The cancer patient population was divided into two groups, tobacco-related cancers and all other cancers. Tobacco-related cancers included: lung, urinary bladder, oral, pharyngeal, head and neck, and laryngeal cancers. Univariate analysis comparing tobacco-related cancers and all other cancers were conducted with regards to age at diagnosis, age groups (0–25, 26–50, 51–75, 76+), sex, smoking status, disease stage, and rural/urban status, with a p-value of 0.05 considered significant. For all tobacco-related cancers and for some sub-groups, such as lung; head, neck, and oral; and urinary and bladder cancer categories, we calculated world age-standardized incidence rates for the whole period of 1998 – 2008 using the WHO 2000–2025 Standard for each sub-district [26]. “World age-standardized rates” are rates that were adjusted using the world population as the reference population. In addition, world age-standardized incidence rates were calculated for each gender and compared by rural and urban case location. The age standardized rates for selected cancers were visualized by sub-district, to show geographic variation in cancer types. All statistical analyses were conducted using SAS 9.4 (Cary, NC) and maps were created using ESRI’s ArcMap 10.2 (Redlands, CA).

## Results

There were 22,415 patients in the CyCR during 1998–2008. Of these, 3,635 patients were diagnosed with tobacco-related cancers and 18,780 with other cancers (Table 1). We included cancers of lung, urinary bladder, oral pharyngeal, head and neck, and laryngeal cancers in the tobacco-related cancers group. Patients diagnosed with tobacco-related cancers were older than their non-tobacco related counterparts (mean age 67.2 ± 12.4 vs. 62.4 ± 17.1, *p* < 0.0001). The majority of patients in the tobacco-related (63.4 %) and all other cancers (55.2 %) were in the 51–75 age group. Male gender was associated with the presence of tobacco-related cancers (OR 4.82, 95 % CI: 4.43-5.26). Among those with tobacco-related cancers, 80.1 % were male compared to 45.4 % males among those with other cancers. A higher proportion of patients who were ever smokers (current or former smokers) were found among patients with tobacco-related cancers (47.3 %) compared to patients with other cancers (19.8 %), p-value < 0.0001 (Table 1). Although the proportion of ever smokers was higher among males compared to females in both urban and rural districts in the general population (Fig. 1), the association was stronger in rural areas compared to urban (OR 13.4, 95 % CI: 8.44-21.28 vs. OR 9.4, 95 % CI: 6.83-12.23) (data not shown).

**Table 1:** Comparison of characteristics between participants diagnosed with tobacco-related cancer compared to all other cancers

Characteristics	Other cancers		Tobacco-related Cancers^1^		*p*-value
	N = 18,780	%	N = 3,635	%	
*Age at Diagnosis*					
Mean Age (StD)	62.4 (17.1)		67.2 (12.4)		<0.0001
Median Age	65.0		68.0		
Range	0-107		4-100		
IQR	53-75		60-76		
*Sex*					
Male	8526	45.4	2910	80.1	<0.0001
Female	10254	54.6	725	19.9	
*Smoking Status*					
Ever smoker	3719	19.8	1721	47.3	<0.0001
Never smoker	5428	28.9	471	13.0	
Unknown	9633	51.3	1443	39.7	
*Stage*					
Local	7584	40.4	1630	44.8	<0.0001
Regional	4472	23.8	596	16.4	
Distant	3219	17.1	669	18.4	
Unstaged	3505	18.7	740	20.4	
*Location*					
Urban	13015	69.3	2476	68.1	0.1563
Rural	5765	30.7	1159	31.9	

Notes:

^1^Tobacco-related cancers include: lung, urinary bladder, oral, pharyngeal, head and neck, and laryngeal cancers

In addition, stage of diagnosis varied by cancer diagnosis group (p-value <0.0001). Among patients with tobacco-related cancers, the majority (44.8 %) were diagnosed at local stage, followed by 18.4 % at distant stage. Among patients with other cancers, 40.4 % were diagnosed at local stage and 23.8 % at regional stage.

Tobacco-related cancers were further classified into lung, head and neck/oral, and urinary bladder cancers. Head and neck/oral cancers included lip, tongue, mouth, salivary glands, tonsil, other oropharynx, nasopharynx, hypopharynx, pharynx unspecified, nose/sinuses, and larynx.

**Fig. 2:**
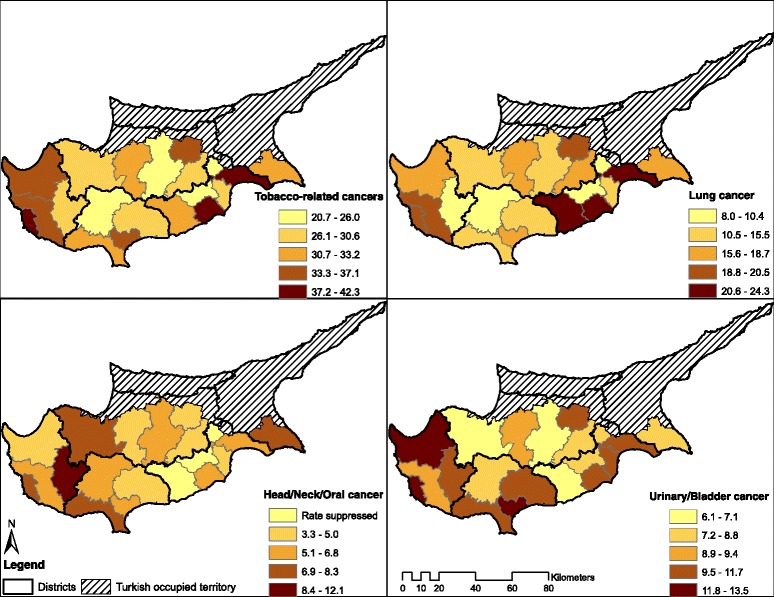
Age-adjusted cancer incidence rates (per 100,000 persons) by Sub-District for all tobacco-related cancers combined, lung cancer, head/neck/oral cancer, and urinary/bladder cancer

There were significant geographic variations among these cancer types by sub-district (Table 2 and Fig. 2). For example, the age adjusted rate (AAR) for tobacco-related cancers was the highest for sub-district 41 in Larnaka (AAR 41.9, 95 % CI: 35.7-48.1), sub-district 60 in Pafos (AAR 40.3, 95 % CI: 35.2-45.3), and sub-district 50 in Lemesos (AAR 36.3, 95 % CI: 33.8-38.7). The age-adjusted tobacco-related cancer incidence rate was the lowest for sub-district 42 in Larnaka (AAR 20.7, 95 % CI: 13.3-28.0) and sub-district 53 in Lemesos (AAR 24.6, 95 % CI: 19.3-29.9). Furthermore, the age adjusted incidence rate of lung cancer was the highest in sub-district 41 in Larnaka (AAR 23.8, 95 % CI: 19.2-28.5). Also, the rates of both head and neck/oral and lung cancer were the highest in Pafos: AAR 12.1 (95 % CI: 1.5-22.7) in sub-district 62 and AAR 13.5 (95 % CI: 10.5-16.4) in sub-district 60, respectively.

**Table 2:** Age-adjusted rates (per 100,000 persons) of tobacco-related cancers by Sub-District

District	Sub-District	All Tobacco-related Cancers		Lung		Head and Neck/Oral^a^		Urinary/Bladder	
		AAR	95 % CI	AAR	95 % CI	AAR	95 % CI	AAR	95 % CI
Lefkosia	10	34.5	32.4-36.6	18.9	17.4-20.5	4.3	3.6-5.1	11.2	10-12.4
	11	28.9	22.1-35.7	17.5	12.2-22.8	3.3	1.0-5.5	8.1	4.6-11.7
	12	25.5	20.9-30.2	13.2	9.9-16.6	6.2	3.8-8.6	6.1	3.8-8.4
	13	31.2	24.3-38.1	17.2	12.2-22.2	4.6	1.8-7.5	9.4	5.5-13.2
	14	28.4	21.3-35.5	14.1	9.3-18.8	7.4	3.0-11.8	6.9	3.6-10.2
	Total	33.1	31.3-34.8	18.4	17.1-19.7	4.5	3.9-5.2	10.1	9.2-11.1
Ammochostos	31	32.2	27.3-37.2	16.3	12.8-19.9	7.2	4.8-9.6	8.7	6.1-11.2
Larnaka	40	30.0	26.7-33.3	15.0	12.6-17.3	4.9	3.5-6.2	10.1	8.2-12.1
	41	41.9	35.7-48.1	23.8	19.2-28.5	6.4	3.9-8.8	11.7	8.4-14.9
	42	20.7	13.3-28.0	9.7	4.6-14.8	RS^b^	RS^b^	8.1	3.5-12.7
	43	31.6	21.5-41.6	21.9	13.2-30.7	RS^b^	RS^b^	7.1	2.9-11.3
	Total	32.3	29.7-35.0	17.3	15.3-19.2	4.9	3.8-6.0	10.2	8.7-11.6
Lemesos	50	36.3	33.8-38.7	17.8	16.1-19.5	5.5	4.5-6.5	13.0	11.5-14.5
	51	28.6	22.1-35.1	14.8	10.2-19.5	3.7	1.3-6.0	10.1	6.3-14.0
	52	32.6	25.6-39.7	13.7	9.2-18.2	8.3	4.7-12.0	10.6	6.7-14.5
	53	24.6	19.3-29.9	9.0	5.8-12.1	6.8	3.8-9.9	8.8	5.8-11.8
	Total	35.0	32.9-37.1	17.2	15.7-18.6	5.7	4.8-6.5	12.1	10.9-13.3
Pafos	60	40.3	35.2-45.3	19.3	15.8-22.8	7.5	5.3-9.7	13.5	10.5-16.4
	61	33.8	26.4-41.2	17.7	12.4-23.1	6.7	3.3-10.1	9.3	5.4-13.2
	62	30.6	18.1-43.0	8.0	2.5-13.5	12.1	1.5-22.7	10.5	4.0-17.0
	63	35.3	26.2-44.5	17.9	11.4-24.4	RS^b^	RS^b^	13.1	7.9-18.4
	Total	36.7	33.2-40.3	18.3	15.8-20.8	6.7	5.1-8.3	11.7	9.7-13.6

^a^Includes: Lip, tongue, mouth, salivary glands, tonsil, other oropharynx, nasopharynx, hypopharynx, pharynx unspecified, nose/sinuses, larynx

^b^Rates were suppressed (RS) if less than 5 cases were reported in a sub-district

The age-adjusted incidence rates for all tobacco-related cancers were higher in males than in females, independent of urban/rural living status. For lung and urinary/bladder cancers rates were higher in urban compared to rural areas, while head and neck/oral cancers were lower in urban compared to rural areas (Table 3, Fig. 2). The largest difference between urban and rural areas were found in females with lung cancer (RR 1.4) and males with urinary/bladder cancer (RR 1.4). The overall tobacco-related cancer rate was the highest among males in the urban districts (AAR 60.8, 95 % CI: 58.2-63.5). Males from the urban areas had the highest lung cancer (AAR 30.9, 95 % CI: 29.0-32.8) and urinary/bladder cancer age-adjusted incidence rates (AAR 21.6, 95 % CI: 20.0-23.2) (Table 3).

**Table 3:** Age-adjusted rates (per 100,000 persons) for urban and rural residence for tobacco-related cancers

Cancer location	Overall AAR		Urban AAR		Rural AAR		Rate Ratio Urban:Rural	
	AAR	95 % CI	AAR	95 % CI	AAR	95 % CI	RR	95 % CI
Tobacco-related: All	33.9	32.8-35.0	35.4	34.0-36.8	31.4	29.6-33.2	1.1^a^	1.1-1.2
Males	57.9	55.8-60.0	60.8	58.2-63.5	53.2	49.7-56.6	1.1^a^	1.1-1.2
Females	13.0	12.0-13.9	13.6	12.4-14.8	11.9	10.3-13.4	1.1	1.0-1.9
Lung	17.8	17.0-18.6	18.6	17.6-19.6	16.5	15.2-17.8	1.1	1.0-1.2
Males	29.9	28.4-31.5	30.9	29.0-32.8	28.6	26.1-31.1	1.1	1.0-1.2
Females	7.3	6.5-8.0	8.0	7.1-9.0	5.8	4.7-6.8	1.4^a^	1.1-1.7
Head and Neck/Oral^b^	5.3	4.8-5.7	5.1	4.5-5.6	5.8	5.0-6.6	0.9	0.7-1.1
Males	8.5	7.7-7.7	8.3	7.3-9.3	8.8	7.4-10.3	0.9	0.8-1.2
Females	2.4	2.0-2.8	2.2	1.7-2.7	2.9	2.1-3.8	0.7	0.5-1.1
Urinary/Bladder	10.8	10.2-10.2	11.8	11.0-12.6	9.1	8.1-10.0	1.3^a^	1.1-1.5
Males	19.5	18.2-18.2	21.6	20.0-23.2	15.8	13.9-17.6	1.4^a^	1.2-1.6
Females	3.3	2.9-2.9	3.4	2.8-4.0	3.2	2.4-3.9	1.1	0.8-1.5

^a^Rate Ratio is statistically significant (*p* < .05)

^b^Includes: Lip, tongue, mouth, salivary glands, tonsil, other oropharynx, nasopharynx, hypopharynx, pharynx unspecified, nose/sinuses, larynx

Tables 4 and 5 examine the differences in daily smoking prevalence for a number of European countries, and compare them to the prevalence of lung and bladder cancers. Among males 65–69 years old (Table 4), only Latvia had a higher smoking prevalence than Cyprus in 1990; in 2000, Denmark, Estonia, Lithuania, and Latvia had higher proportions of daily smokers than Cyprus. However, with regard to cancer, all countries except for Sweden exhibited a higher lung cancer rate than Cyprus. For bladder cancer, seven countries exhibited higher rates than Cyprus, despite having similar or lower smoking prevalence. Table 5 examines the smoking and cancer rates among 65–69 year-old females. There were 12 countries that had a higher smoking prevalence than Cyprus in 1990 and/or 2000; these countries also had higher lung and/or bladder cancer rates.

**Table 4:** Smoking prevalence among males ages 65–69 years in 1990 and 2000 compared to cancer rates for adults ages 15+ (1998–2007)

Country	Proportion of male daily smokers % (UI)		Lung^a^	Bladder^a^
	1990	2000		
**Cyprus**	**32 % (17-41 %)**	**32 % (20-47 %)**	**29.9**	**19.5**
Sweden	22 % (18-27 %)	17 % (14-21 %)	20.8	17.4
Slovenia^b^	19 % (9-32 %)	16 % (9-26 %)	57.7	16.7
Finland^b^	15 % (11-20 %)	13 % (11-16 %)	35.8	13.9
Malta^b^	27 % (14-44 %)	24 % (15-34 %)	36.8	25.5
Slovakia ^b^	11 % (5-20 %)	6 % (3-10 %)	58.7	15.4
Denmark^c b^	29 % (25-34 %)	34 % (30-39 %)	45.6	26.4
Netherlands^b^	27 % (24-31 %)	17 % (14-21 %)	52.0	14.2
United Kingdom^b^	25 % (22-29 %)	20 % (18-22 %)	42.9	21.1
Germany^b^	28 % (23-34 %)	18 % (15-22 %)	58.9^d^	19.0^d^
Italy^b^	29 % (25-34 %)	21 % (18-24 %)	53.2	33.9
Austria^b^	24 % (17-31 %)	22 % (16-28 %)	44.2	22.5
France^b^	26 % (22-31 %)	17 % (13-21 %)	52.5	19.3
Czech Republic^b^	20 % (10-32 %)	22 % (16-28 %)	61.5	20.0
Estonia^cb^	32 % (18-49 %)	36 % (23-50 %)	60.8	16.0
Spain^b^	28 % (24-34 %)	24 % (20-28 %)	49.2	34.8
Lithuania^cb^	26 % (14-43 %)	36 % (20-52 %)	61.3	16.4
Latvia^cb^	37 % (22-56 %)	44 % (29-58 %)	62.0	15.4

*UI* Uncertainty intervals

^a^Per 100,000. Cancer rates for Cyprus are derived from the present study. Cancer rates from all other countries are from CI5 (1998–2007)

^b^These countries had higher lung and/or bladder cancer rates than Cyprus

^c^These countries had a higher smoking prevalence than Cyprus for 1990 and/or 2000

^d^Only includes the German state of Saarland

**Table 5:** Smoking prevalence among females ages 65–69 years in 1990 and 2000 compared to cancer rates for adults ages 15+ (1998–2007)

Country	Proportion of female daily smokers % (UI)		Lung^a^	Bladder^a^
	1990	2000		
**Cyprus**	**6 % (2-14 %)**	**7 % (3-14 %)**	**7.3**	**3.3**
Sweden^bc^	18 % (12-23 %)	15 % (12-19 %)	15.9	5.0
Slovenia^bc^	8 % (3-17 %)	7 % (3-13 %)	14.2	3.8
Finland^bc^	6 % (4-9 %)	8 % (6-10 %)	10.2	2.9
Malta^bc^	8 % (3-17 %)	6 % (3-11 %)	6.4	5.3
Slovakia^c^	5 % (2-11 %)	5 % (2-9 %)	9.4	3.5
Denmark^bc^	33 % (28-39 %)	30 % (25-34 %)	33.4	7.7
Netherlands^bc^	14 % (11-17 %)	14 % (11-18 %)	22.6	3.4
United Kingdom^bc^	24 % (20-29 %)	19 % (17-22 %)	24.6	6.1
Germany^bc^	10 % (7-14 %)	10 % (7-12 %)	19.2^d^	5.1^d^
Italy^bc^	12 % (9-15 %)	10 % (8-11 %)	12.7	6.2
Austria^bc^	9 % (6-14 %)	12 % (8-17 %)	15.4	5.7
France^bc^	10 % (8-13 %)	9 % (6-12 %)	11.1	2.9
Czech Republic^bc^	14 % (6-26 %)	12 % (8-18 %)	14.9	5.3
Estonia^c^	4 % (2-10 %)	5 % (2-11 %)	8.8	2.9
Spain^c^	6 % (4-8 %)	4 % (3-5 %)	6.0	4.5
Lithuania	2 % (1-5 %)	2 % (1-5 %)	6.6	3.0
Latvia^c^	5 % (2-11 %)	6 % (3-12 %)	7.7	2.7

*UI* Uncertainty intervals

^a^Per 100,000. Cancer rates for Cyprus are derived from the present study. Cancer rates from all other countries are from CI5 (1998–2007)

^b^These countries had a higher smoking prevalence than Cyprus for 1990 and/or 2000

^c^These countries had higher lung and/or bladder cancer rates than Cyprus

^d^Only includes the German state of Saarland

Similar patterns in smoking prevalence and cancer rates were found for the same countries among 60–64 year-old adults. Latvia was the only country that exhibited a higher proportion of male daily smokers than Cyprus (for the year 2000), however every country except for Sweden exhibited higher rates of lung cancer. Additionally, six countries exhibited higher rates of bladder cancer than Cyprus despite lower proportions of daily smokers (data not shown).

## Discussion

This study revealed the following interesting observations. First, tobacco-related malignancies were more common among older males and ever smokers compared to non-tobacco-related malignancies. Second, there were distinct variations in AARs of tobacco-related malignancies for the sub-districts of Cyprus. Third, we found that age-adjusted incidence rates were also at least three times as high for males compared to incidence rates among females for all tobacco-related cancers (lung, head/neck/oral, and urinary bladder). Furthermore, among tobacco-related cancers, lung cancer had the highest overall AAR while head and neck/oral cancer had the lowest overall AAR. Lastly, tobacco-related cancer rates for Cyprus in this study were lower than the rates of the same cancers reported from other countries.

The large proportion of tobacco-related cancers in males compared to females observed in this study is supported by the higher number of Cypriot men who reported smoking than women in the 2003 and 2008 health surveys [17]. A previous meta-analysis of 254 studies also found that among various cancer sites, male smokers had higher relative risks for developing respiratory cancers than females [27]. Additionally, a larger proportion of tobacco-related cancers were in patients between the ages of 51 and 75 compared to non-tobacco related cancers. This could be due to the long lag period needed for the development of tobacco-related malignancies following tobacco exposure [23, 28, 29].

The highest AARs of all tobacco-related cancers were found in sub-district 41 of Larnaka, followed by sub-districts 60 of Pafos and 50 of Lemesos. However, Larnaka and Lemesos were also home to the lowest AARs of all tobacco-related cancers (in sub-districts 42 and 53, respectively). The intra- and inter-district variations are likely to be influenced by the proportion of rural and urban Cypriot smokers. As of 2008, nearly one-third of the Cyprus urban population reported smoking, as shown in Fig. 1, while rural areas reported smaller percentages [18]. The observed high number of urban smokers coincides with the finding from our study that the highest rates of cancer were in sub-districts 60 and 50, both of which are urban. It is possible that although sub-district 41 is rural, residents are more exposed to environmental risks than neighboring sub-districts, thereby leading to increased cancer rates within the sub-district. Such environmental risks include chemical exposures from farming and the proximity to an oil refinery that was active up to a few years ago. It is also possible that residents from sub-district 41 smoke more frequently than others from neighboring sub-districts; however, data on smoking prevalence in each sub-district is not available. Further studying is needed to identify what gives rise to the high rate of cancer in a rural sub-district.

The observations regarding sub-districts 60 and 50 were further supported when the data was stratified by urban versus rural populations of Cyprus. The AAR for all tobacco-related cancers combined was higher in urban compared to rural populations. While multiple factors, such as the environment or occupational exposures, are related to the urban and rural differences in cancer rates, the clearly higher prevalence of smoking in urban areas is likely an important factor [30, 31]. A smoking pattern similar to what is shown in Fig. 1 was found in Greece, where marked differences in smoking between rural and urban residents of similar age ranges and educational status were observed [32]. Other European countries, such as Poland, also reported higher proportions of smokers in urban (nearly one-third) compared to rural areas (about one-fourth) [31].

It should be noted that the AAR for head and neck cancer found in our study was higher for rural than urban populations. This could be due to a number of factors, such as differences in alcohol consumption, infections, or nutritional deficiencies. Although interesting, the exact cause of this observation is unknown and should be further examined.

In our study, cancer rates were higher in males than females in the lung, head and neck, and urinary/bladder in both rural and urban settings. Again, a main factor contributing to this gender difference in cancer rates could be the greater number of men who smoke compared to women [17, 33]. The 2008 European Health Survey found that, on average, men smoked 24 cigarettes per day whereas women smoked an average of 15 cigarettes per day [18]. There may also be gender differences in the additional risk factors previously mentioned that contribute to the development of cancer. These should be taken into consideration in future studies.

Among the different cancer sites, the highest overall AAR was found for lung cancer whereas the lowest was for head and neck. Although a causal relationship exists between smoking and both cancer sites, it is interesting that one anatomic site showed higher rates of cancer than the other, despite both being exposed to tobacco during smoking or other methods of tobacco inhalation. A possible reason for this rate difference is the high rate of cell-turnover of epithelial cells in the oral cavity and esophagus, and the higher susceptibility to damage of alveolar cells in the lungs [34, 35]. For other tobacco-related cancer sites exposure to the effect of smoking is secondary. For example, bladder cells are exposed to carcinogens that accumulate in the urine after they have been carried through the bloodstream, whereas cells of the lungs are directly exposed to tobacco smoke [36, 37].

Another factor that could give rise to the different incidence rates of cancer in various sites is the type of tobacco product and inhalation pattern. The smoke produced from pipes and cigars irritate and damage the pharynx, as opposed to damaging the alveoli of the lungs [29]. Conversely, smokers inhale more deeply when using cigarettes, thereby increasing the concentration of smoke and carcinogens within their lungs [29]. According to the 2008 Health Survey, the prevalence of cigarette smoking among male smokers was 38.9 %, while the prevalence of cigar and pipe smoking was only 1.4 %. For females, the prevalence of cigarette smoking was 14.4 % and there was no reported cigar or pipe smoking [18].

Although there is a strong causal relationship between smoking and the various cancer sites studied, there are also other risk factors associated with the development of disease, which may be less prevalent in Cyprus compared to other countries. These include genetic polymorphisms, infections (e.g. human papillomavirus), ionizing radiation, organic chemicals, air pollution and occupational exposures [23, 29, 38–40]. For example, in Romania, which has much higher lung cancer rates than Cyprus, the population is exposed to radon gas in dwellings. A study estimated that in two different counties in Romania (Cluj and Bistrita), 9.09 % and 5.66 % of lung cancers in non-smokers were attributed to radon, respectively [41]. Thus, information regarding different exposures in cancer patients is imperative to understanding the etiology of their disease. In addition, future investigations should explore the histological subtypes of lung cancer in these populations with different lung cancer incidence. Another interesting potential difference that is worth investigating in the future relates to the types of tobacco smoked in these different countries. Finally, there is a possibility that differential screening and detection among these countries may affect the reported incidence of these cancers, even though Cyprus along with these other countries follow the European guidelines, which recommend against lung cancer screening [42, 43].

Despite the high prevalence of smoking within Cyprus, the observed cancer rates for the country are lower than the rates in other European countries, especially for males. It is possible that wind on the island could help move smoke in the atmosphere, which could contribute to lower cancer rates. This pattern of high smoking rates paired with low tobacco-related cancer rates has also been observed in Japan and aptly called the “Japanese smoking paradox” [44]. The factors (genetic and/or environmental) that contribute to this observation are unknown and should be further studied.

Strengths of this study include the large dataset used from the Cyprus Cancer Registry, the dataset’s previous utilization in research and comparisons with other countries [20, 26], and the use of maps depicting all geographic regions of Cyprus. However, the ecologic nature of the study and lack of information regarding measurements of individual exposures to tobacco limited our ability to draw conclusions after hypothesis generation. Therefore, future studies should focus on measuring individual exposures to tobacco smoke and understanding smoking behaviors in addition to other possible etiologic factors in the development of tobacco-related malignancies.

## Conclusions

In conclusion, this study illustrates higher rates of tobacco-related cancers in males than females in Cyprus. The study also reveals geographic differences between districts and sub-districts and differences in incidence between individual types of tobacco-related cancers. The study illustrates lower incidence tobacco-related cancers in Cyprus compared to other countries with similar smoking rates. Future studies should be in-depth with individual measurements of different types of smoking and account for other risk factors of tobacco-related malignancies. Since tobacco-related cancer rates in Cyprus are low despite high smoking prevalence, understanding the factors that contribute to the lower cancer incidence will have important implications for cancer prevention. These factors could be genetic and/or environmental and their identification is essential in order to reduce tobacco-related cancer incidence in other populations. Furthermore, although tobacco-related cancer rates are low within Cyprus, smoking cessation should still be emphasized to further reduce the burden of cancer and other side effects related to smoking.
